# Supplementary material for: Medical Education: Patients’ Perspectives on Clinical Training and Informed Consent
Source: Int J Environ Res Public Health. 2022 Jun 22;19(13):7611. doi: 10.3390/ijerph19137611 (PMC9265405; doi:10.3390/ijerph19137611)
Supplement: Supplementary file 1 [file ijerph-19-07611-s001.zip › ijerph-1741201-supplementary.pdf]

MEDICAL EDUCATION: PATIENTS' PERSPECTIVES

Please complete the following questionnaire by selecting the appropriate boxes or writing down the required information.

1. DEMOGRAPHIC DATA

1. Gender:
- **i.** Male ___
- **ii.** Female ___

2. Age: _______ years

3. Marital status:
- i. Single ___
- ii. Married ___
- iii. Widowed ___
- iv. Divorced ___
- v. Joint union ___

4. Academical achievements:
- i. Didn't attend school ___
- ii. Didn't complete primary school ___
- iii. Primary school ___
- iv. Middle school ___
- v. Secondary school ___
- vi. Tertiary education ___
- vii. Other classification: ___________________________

5. Household:
- i. Husband/wife/partner _____
- ii. Children. How many? _____
- iii. Siblings. How many? _____
- iv. Nephews. How many? _____
- v. Others. Which? _____

6. Profession:____

2. QUESTIONS RELATED TO THE PROJECT OF INVESTIGATION

1. Have you ever participated in a medical appointment in which a medical student (or group of medical students) was present?
- i. Yes ___
- ii. No ___

2. If yes, did your doctor ask for your permission for the student(s) to be present?
- i. Yes ___
- ii. No ___

3. Did your doctor introduce the students by name and year of medical education?
- i. Yes ___
- ii. No ___

4. Did you feel unconfortable with the situation? If yes, how much so?
   i. Not at all ___
   ii. Moderately ___
   iii. A lot ___

5. Did the medical student(s) that took part in your medical care introduce themselves as medical students and ask for your consent before they did the medical exam?
   i. Yes ___
   ii. No ___

6. Did the student(s) explain the procedures that they wanted to perform and answered your questions, if you had them?
   i. Yes ___
   ii. No ___

7. When there are medical students present during your medical care do you feel that you get more information/explanations about your illness/condition?
   i. Yes ___
   ii. No ___

8. Was there any occasion in which the student(s) present were disrespectful towards you?
   i. Yes ___
   ii. No ___

9. Do you feel pleased by having contributed to the students' medical education?
   i. Yes ___
   ii. No ___

10. In case your condition was gynecological, urological or any other related to an intimate part of your body, would you feel more bothered by the presence of one or more medical students in your appointment?
   i. Yes ___
   ii. No ___

10.1. IF YES, would you feel comfortable to express your discomfort?
   i. Yes ___
   ii. No ___

11. Are you afraid of revealing an intimate problem during an appointment in the presence of one or more students?
   i. Yes ___
   ii. No ___

12. If you could choose not to have students present during your medical appointment, would you feel more comfortable?
   i. Yes ___
   ii. No ___
